# Controlled release of vitamin D_3_ using a nanocellulose-based membrane

**DOI:** 10.1038/s41598-022-16179-2

**Published:** 2022-07-20

**Authors:** Pedro L. Colturato, Danielle Goveia

**Affiliations:** 1 Department of Bioprocess and Biomaterial Engineering, Faculty of Pharmaceutical Sciences, State University of São Paulo (UNESP), Araraquara, Itapeva, SP 18409-010 Brazil; 2 Itapeva Experimental Campus, State University of São Paulo (UNESP), Itapeva, SP Brazil

**Keywords:** Biotechnology, Drug discovery, Environmental sciences, Nanobiotechnology

## Abstract

Epidemiological studies show that a significant fraction of the global population presents low levels of vitamin D_3_. In order to address this problem, one way to administer the vitamin is to incorporate it in novel drug delivery systems, such as transdermal devices. A possible substance for this purpose is cellulose, which has a long history of use in the health area. However, the application of nanostructured cellulose membranes, as local drug delivery systems, remains a challenge. To develop a crystalline nanocellulose membrane as a new tool for the release of vitamin D_3_. A new nanostructured membrane containing nanocellulose extracted from cotton linter and vitamin D_3_ was produced using the “casting” technique. The membrane was characterized using high-resolution scanning electron microscopy (FEG-SEM) and Fourier transform infrared spectroscopy (FT-IR). The kinetics of vitamin release was quantified using molecular spectroscopy (UV–Vis). The FT-IR spectra showed the presence of all the active components in the membrane sample, without structural alterations or the formation of new bonds. The FEG-SEM images showed the presence of vitamin crystals on the surface and in the interior of the membrane. The release of vitamin D_3_ occurred in a sustained manner, obtaining 3029 IU mL^−1^ of vitamin D_3_ in 60 min. The findings demonstrated that the membrane could be used for the sustained release of vitamin D_3_. This new biomaterial has potential as a new model for vitamin supplementation in individuals with vitamin D_3_ deficiency.

## Introduction

Vitamin D_3_ (cholecalciferol) is a steroid hormone produced by the photolytic action of UV-B radiation on the 7-dehydrocholesterol molecule found in the epithelial tissue, producing pre-vitamin D_3_, which is absorbed and then metabolized in the liver to 25-hydroxyvitamin D_3_. The latter subsequently reaches the renal tubules and is converted into the active form, 1,25-dihydroxyvitamin D_3_^1–4^. Evidence obtained in the laboratory indicates that the active hormonal form of vitamin D_3_ induces a range of extra-skeletal biological responses, in addition to its effects in bone metabolism, including regulation of skin cell proliferation, effects in the cardiovascular system, and protection against various autoimmune diseases, multiple sclerosis, cancer, obesity, and inflammatory intestinal disease^5,6^.

More recently, vitamin D_3_ has received increasing attention, because deficiency of this hormone has led to increasing cases of rickets, making it a global health issue. Epidemiological studies have found low levels of this vitamin throughout the global population, irrespective of age, level of education, or race^2,7^.

The three main factors involved in maintaining satisfactory levels of vitamin D_3_ are adequate consumption of foods rich in cholecalciferol, sufficient exposure to solar UV-B to permit transdermal synthesis of the hormone, and dietary supplementation^6^. Populations that present inadequacy of one or more of these sources of vitamin D_3_ are at risk of deficiency^8^. A difficulty is that oral ingestion methods can have limitations in individuals with intestinal disorders, Crohn’s disease, or chronic hepatic, gastric, and renal disorders. In these individuals, the intestinal absorption of this vitamin is compromised^9,10^.

The main risk factors for decreased serum levels of vitamin D_3_ include excessive use of sunscreens, living in closed environments, chronic use of drugs that reduce serum cholesterol and consequently decrease levels of 7-dehydrocholesterol in the skin, advanced age, distance from the equator, air pollution, black skin, smoking, and intestinal malabsorption syndromes, among others^3,6,11^.

Vitamin D_3_ has low water solubility, so various formulations have been developed to increase its solubility, such as the use of liposomes, lipid emulsions, and mixed micelles^12–14^. As an alternative to oral administration of vitamin D_3_, which may present low efficiency due to the various barriers and different conditions encountered, application to the skin can offer advantages such as the treatment of psoriasis, atopic dermatitis, and other allergic skin diseases^15^. However, the main challenge in transdermal drug delivery still relies on the impermeable barrier that the stratum corneum represents, as the outermost layer of the skin. A strategy to improve the penetration of molecules in the skin depends on the use of lipid nanosystems^16^.

Nanocellulose (NC) has a long history of applications in the pharmaceutical industry, as excipients, functioning as special mediators in the controlled and prolonged release of drugs in tablets, mainly due to its disintegration rate. In this context, the physicochemical forms of nanocellulose-based drug carriers can be divided into 3 groups: (i) microspheres (or microparticles). (ii) hydrogels (or gels) and (iii) membranes (or biofilms)^17–19^.

The use of NC, whether in the form of bacterial cellulose, nanofibrillated cellulose or cellulose nanocrystals, has been widely explored to produce drug delivery systems, including membranes, for transdermal and topical delivery of different drugs, such as local anesthetics, vitamins, antibiotics, anti-inflammatories, contraceptives, among others. In addition, several strategies have been described to improve the incorporation of lipophilic drugs into nanocellulose-based membranes^14,19–21^.

Currently, new drug delivery systems are being developed to minimize the side effects and increase the therapeutic effect of drugs due to their cutting-edge properties, such as minimal application pain, prevention of first-pass metabolism, prolonged action and its unique ability to provide therapeutic efficacy in the minimum number of dosages. The final challenge of these researches lies in the selection of appropriate excipients and polymers that are non-toxic and biocompatible and that can maintain their activity with minimal undesirable side effects^14,16,21,22^. Materials based on NC have the potential for use in the sustained release of cosmetics and drugs^17,23^.

Controlled release of vitamin D3 may have the advantage of increased elimination half-life and/or reduced toxicity and/or improved potency, e.g., ability to administer a reduced dose of vitamin D compound, or to administer less frequently, to obtain a therapeutic effect compared to an immediate-release dosage^13,24,25^.

PVA presents excellent results in drug delivery systems mainly because it is a highly crystalline, hydrophilic, miscible, biocompatible, non-toxic, biodegradable polymer with good film-forming capacity, in addition to containing massive hydroxyl groups in its macromolecular chains, which can be a source of hydrogen bonds with cellulose. Used as a skin permeation agent, probably due to the interaction of this polymer with the stratum corneum of the skin, it has action as a carrier of active principles. PVAL emerges as a substance with high potential for drug release, since it can assist in the release of compounds in the epidermis^26^.

In fact, the composites formed by the association of PVA and reinforced with nanocellulose currently developed have high mechanical strength and elastic properties and, therefore, have high resilience, being able to withstand large deformations when stretching and recovering their original shape and dimensions when releasing this tension. . Furthermore, the casting method is one of the most attractive techniques for the preparation of PVA membranes, as it allows to obtain 3D structures, devoid of the harmful effect of an additional chemical crosslinker. Such properties make them a good candidate in transdermal matrix film design^25–28^.

Several drug delivery systems require surfactant properties, due to the lipophilic and hydrophobic character of their membrane constituents, the most widely recommended are non-ionic surfactants with a relatively high hydrophilic-lipophilic balance^27^. Previous cytotoxicity studies revealed that non-ionic surfactants such as tween 80 have less toxic effect than cationic, anionic and amphoteric surfactants. Due to its less cytotoxic properties, this surfactant was selected for this study. Using an adequate concentration of surfactant the cumulative release and release rate of the drug can be controlled^28^.

The market for nanocellulose was estimated to be worth US$250 million in 2019, while the number of scientific articles concerning the use of nanocellulose in medical and pharmaceutical applications has steadily increased over the last 10 years^29^. However, there is still a vast field open for the development of new innovations, such as using different functionalities and interactions of bioactive agents, employing functional nanocelluloses for the development of new drug delivery systems and cell culture media, as well as for tissue engineering and for combating bacteria and viruses^30^.

Meanwhile, the number of such products marketed in the medical and pharmaceutical areas remains low, which can be attributed to challenges such as the development of formulations incorporating highly lipophilic or high molar mass drugs, stability during storage, quality control of the drugs, and other factors such as limited production for large batches. It is expected that nanomaterials will find uses in cosmetic products such as lotions, soaps, and skin treatments, as has been discussed at conferences and can be seen in the patent literature. Cosmetics companies have shown interest in bio-based nanomaterials that are biocompatible and biodegradable^31^.

Many investigations have already been undertaken by researchers and companies, with the aims of optimizing the production and marketing of nanocellulose. Nanocellulose has a wide range of applications in the health area, which could contribute to addressing the multiple challenges of modern society. It could have significant future contributions in materials engineering and the attainment of global sustainable development goals^32^.

The aim of the present work was to develop an innovative system for the sustained release of vitamin D_3_, employing a matrix of crystalline NC extracted from cotton linter. The development of a new nanostructured system for the sustained transdermal release of vitamin D_3_ could enable convenient treatment of various skin conditions, as well as provide appropriate supplementation of this hormone.

In this work, the combination of NC and PVA for the development of membranes is an innovation. The novelty is addressed not only by the hybrid approach, but also by the production process. PVA has a hydrophilic structure, with amphiphilic behavior, which makes it a remarkable polymer with exceptional properties, such as biodegradability, biocompatibility, non-toxicity and non-carcinogenicity. The addition of cotton linter nanocellulose, tween 80 and glycerin for membrane development generates a new design, which could enrich vitamin D3 formulations for easy application in the future.

## Materials and methods

The materials used for preparation of the membranes were as follows: nanocellulose, obtained by acid hydrolysis of cellulose from cotton linter, polyvinyl alcohol (DINÂMICA, batch 94332), vitamin D_3_ (FAGRON, sourced from Zhejiang Garden Biochemical High-Tech, batch C20170603A-5), liquid glycerin (SYNTH), Tween 80 surfactant (DINÂMICA), and ultrapure water (resistivity > 18.3 MΩ.cm).

### Nanocellulose extraction

The extraction of NC was performed by acid hydrolysis of cotton linter cellulose with sulfuric acid (64% w/w), using a ratio of 1:10 (pulp (g): acid (mL)), temperature of 60 ºC, and hydrolysis time of 30 min^33^. After this step, the solution obtained was dialyzed using a 21 mm diameter Servapor membrane. The size of the nanocellulose obtained was determined using high-resolution field emission gun scanning electron microscopy (FEG-SEM)^34^.

### Preparation of vitamin D_3_ solution

The vitamin D_3_ used in this work was supplied in the form of a whitish crystalline powder with a concentration of 40,000,000 IU g^−1^. The amount of vitamin D_3_ is usually presented using two units: (i) micrograms (μg), or (ii) international units (IU), where one microgram is equal to 40 IU^10^. Due to this high concentration, a 1:50 (w/w) pre-dilution was performed. A mass of 1.0 g of vitamin D_3_ was weighed out and solubilized in 49.0 g of absolute ethyl alcohol, resulting in a concentration of 800,000 IU per gram of solution.

### Synthesis of the membranes

Three types of membranes were produced (Table 1), two containing vitamin D_3_ at different concentrations, and a control membrane without addition of the active agent. One membrane, denoted “nanovit”, was produced with a vitamin D_3_ concentration of 8,000 IU mL^−1^. A second membrane, denoted “nanovit super”, was produced with 40,000 IU mL^−1^ of vitamin D_3_. The control membrane was produced by adding glycerin, in the absence of the vitamin.

**Table 1:** Concentrations of vitamin D_3_ in the membranes.

Membranes	Nanocellulose (mL)	PVA 5% (mL)	Glycerin (mL)	Tween 80 (mL)	Vitamin D_3_ (IU mL^−1^)
Nanovit	15	50	30	5	8000
Nanovit super	15	50	30	5	40,000
Control	15	50	30	5	No addition

Liquid glycerin was used to solubilize the vitamin D_3_ and as a plasticizer in the mixture, in order to promote membrane formation^35^. For synthesis of the membranes containing the active agent, two liquid glycerin solutions were prepared, with different concentrations of the vitamin. For the first, 1.0 g of the solution of vitamin D_3_ diluted 1:50 in absolute ethyl alcohol was weighed out and added to 30 mL of liquid glycerin, resulting in 800,000 IU of the vitamin in the solution. For the second solution, 5.0 g of the 1:50 solution of vitamin D_3_ in absolute ethyl alcohol was used, resulting in 4,000,000 IU of the vitamin in the liquid glycerin solution.

The weighing employed an analytical balance with precision of 0.0001 g (model AW 220, MARTE/SHIMADZU).

**Figure 1:**
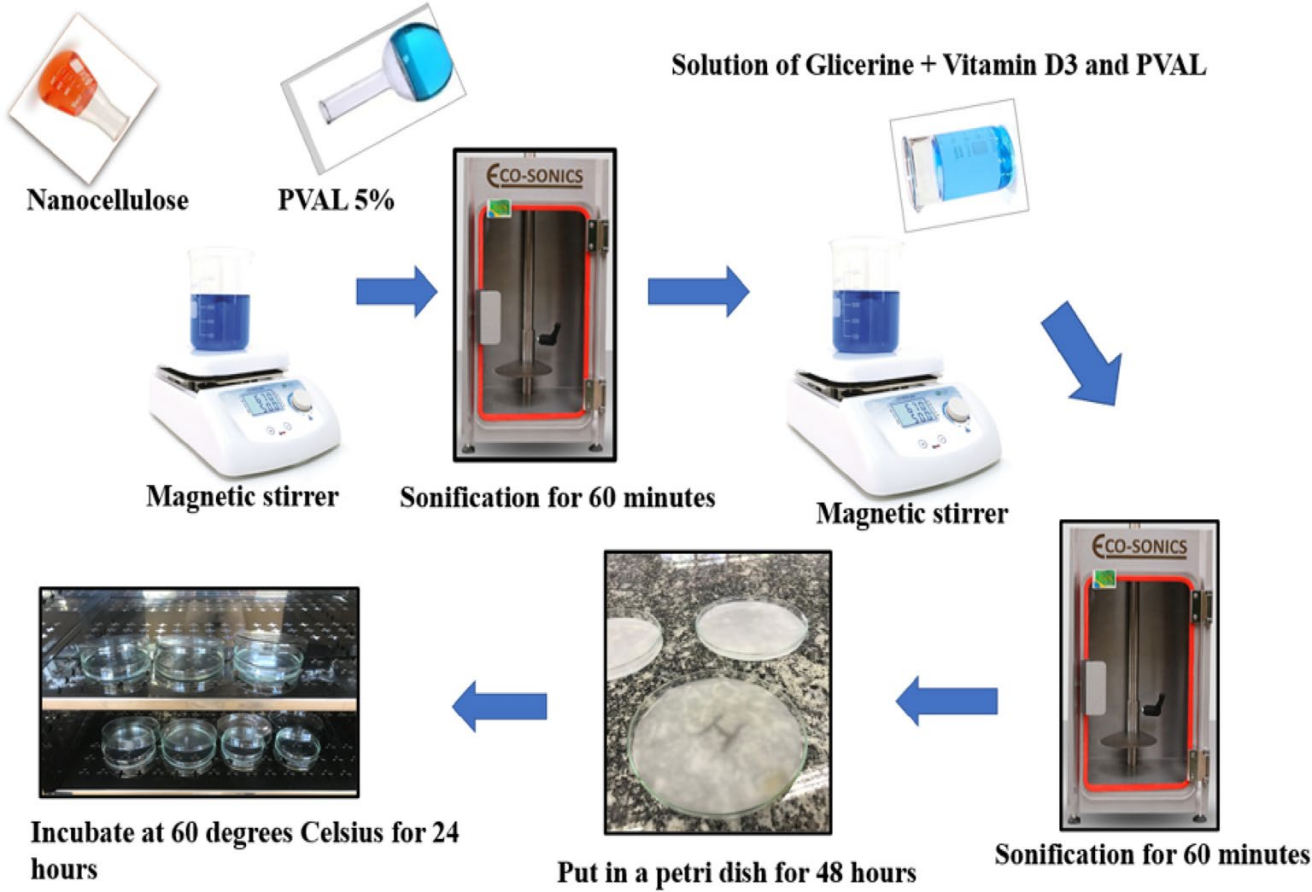
Illustration of the procedure used to produce the nanocellulose membranes containing vitamin D_3_.

As shown schematically in Fig. 1, the components were processed according to the “casting” technique, involving the preparation of a solution capable of forming membranes, following the methodology described by Fakhouri et al.^36^.

A 15 mL volume of nanocellulose was added to 50 mL of 5% PVA solution, with sonication for 60 min to ensure complete dissolution of the nanocellulose, using an ultrasonicator (ULTRONIQUE, Ecosonics) with a 13 mm diameter titanium macrotip, operated at 500 W and frequency of 20 kHz. After these procedures, the PVA and NC solution was mixed with the glycerin solutions containing different concentrations of vitamin D_3_. Tween 80 surfactant was then added to all the solutions, followed by sonication for 60 min.

The solutions were transferred to Petri dishes and left to evaporate at ambient temperature for 48 h, followed by heating in an oven for 24 h at 60 °C.

### Physical characterization of the membranes

The following parameters were considered in evaluation of the physical characteristics of the membranes: mass, thickness, diameter, elasticity, flexibility, tensile strength, and resistance to tearing and crushing^37–39^. The tests were performed in triplicate and the results were reported as means and standard deviations. The temperature during the tests was around 26 °C.

The membranes were divided into two groups: (i) control membranes, without addition of vitamin D_3_, and (ii) nanovit membranes, with addition of vitamin D_3_.

Mechanical traction tests were performed using a QUALITYLABOR dynamometer to measure the stretching and strength of the membranes before and after incorporation of vitamin D_3_^20^.

Cross-sectional area measurements were performed using membrane specimens with standardized dimensions of 60 mm (length), 20 mm (width), and 4 mm (thickness). These measurements were carried out following the dynamometer specifications for the test, using test specimens with standardized cross-sectional area of 80 mm^2^ (width x thickness).

Tear resistance was measured using an Elmendorf model 53984 Digital Tear Tester (FRANK), which uses a pendulum with defined loads to determine the force (kg.f) indicating the tear resistance of a material. Test specimens of the control and nanovit membranes were prepared with dimensions of 100 mm (length), 60 mm (width), and 4 mm (thickness)^40^.

In the crushing test, the compression resistance (kN/m) of the membranes was measured using a Short Span Compression Tester (SCT, model 18510, FRANK). The sample was placed in the open clamps, the device was switched on, and the clamps closed at a rate of 3 ± 0.1 mm/min. The standardized specimen size was 60 mm (length), 20 mm (width), and 4 mm (thickness)^41^.

### Fourier transform infrared spectroscopy (FT-IR)

Spectra of the dried membrane samples were acquired using a Bruker Tensor 27 FT-IR instrument operated in fully attenuated reflection mode, in the range 350–4000 cm^−1^, at a resolution of 4 cm^−1^, with accumulation of 32 scans. The membranes were divided into two groups, without (controls) and with addition of vitamin D_3_ (nanovit membranes)^42,43^.

### High-resolution scanning electron microscopy (FEG-SEM)

A high-resolution field emission electron microscope (model JSM-7500F, JEOL)^34,44,45^ was used to acquire images for evaluation of the control and nanovit membrane surfaces, the surface of the nanovit membrane after release of vitamin D_3_ in absolute ethyl alcohol solution, and the size of the nanocellulose obtained from the cotton linter.

The samples were previously dehydrated, mounted on stubs, and metalized using a BAL-TEC SCD-50 system, followed by coating with carbon, under vacuum^46^. For all the carbon depositions, the distance from the sample was 50 mm and the vacuum was 2 × 10^–1^ mbar. The presence of a conductive carbon layer is usually required to reduce the effect of electrical charge on the surface caused by the interaction between the electron beam and the sample, which can lead to distortion of the image^47^.

### Vitamin D_3_ release kinetics

In the release assays, the quantification of vitamin D_3_ released was performed by ultraviolet–visible (UV–Vis) spectrophotometry, using a SPECORD 50 single-beam spectrophotometer fitted with deuterium (UV) and halogen (Vis) lamps, and a detector for measurement in the range from 190 to 1100 nm. The absorbance readings were obtained with the samples in 1 cm quartz cuvettes. Although the American Pharmacopoeia recommends the use of chromatographic methods for the direct quantification of drugs, spectrophotometric techniques have the advantages of being fast and relatively inexpensive. Hence, the use of spectrophotometry is an attractive option, given standardization of the methodology^48,49^.

The following procedures were performed in the release assays: (i) selection of the wavelength at which vitamin D_3_ presented the highest optical absorbance; (ii) construction of the standard curve for calibration of the optical response of vitamin D_3_, using serial dilutions in the linear range from 0 to 8,000 IU mL^−1^, in absolute ethyl alcohol^50^; (iii) selection of the receptor liquids for release of the vitamin, either a solution containing a mixture of 50% (v/v) absolute ethyl alcohol and 50% (v/v) alkaline phosphate buffer, or a solution of absolute ethyl alcohol alone; (iv) recording of the absorbance peaks for the reagents used in the membrane procedures; and (v) measurement of the release of the vitamin in the receptor liquid and construction of the curve of release as a function of time. The release assays were performed in beakers containing the membranes and 200 mL volumes of the receptor liquids, with 5 mL aliquots of solution being withdrawn after 5, 15, 30, 45, and 60 min, for analysis by UV–Vis spectrophotometry and determination of the concentration of vitamin D_3_ released as a function of time^24^.

## Results and discussion

### Physical characterization of the membranes

Polymers, such as cellulose membranes, show behavior intermediate between elastic materials and high viscosity liquids^51^. The mechanical testing indicated that addition of the vitamin to the polymeric membrane matrix altered the resistance and specific deformation of the material. In principle, the addition of vitamin D_3_ in the bulk membrane could lead to increases of cross-linking and interaction between the chains by van der Waals forces and hydrogen bridges, forming a three-dimensional network within the material^52^.

The formation of crosslinks has a major influence on the mechanical properties of polymeric materials. In the case of sustained release techniques, it is essential that the mechanical characteristics of the materials are well known, since the strengths of the bonds between the components present in the membranes can influence processes such as those involved in the action of local drug release systems. Matrices with crosslinks between the polymer chains may enable membranes to absorb a greater quantity of water without dissolving, so their continuing adhesion to the surfaces on which they are applied can allow prolonged drug release over an extended period^53^.

**Figure 2:**
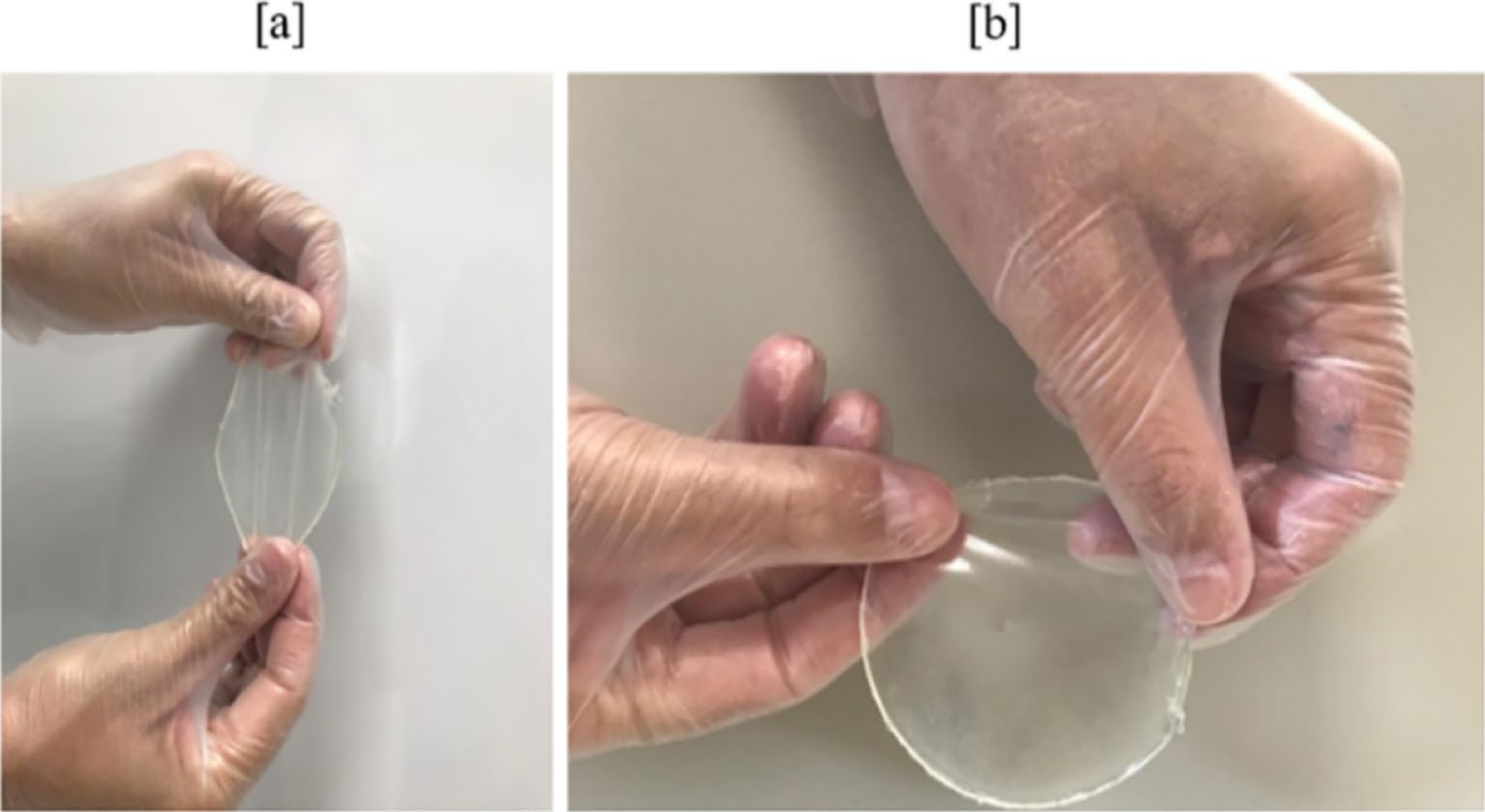
General aspects and visual appearance of the crystalline nanocellulose membranes.

Evaluation was made of the general characteristics of the synthesized membranes. Figure 2 shows the physical aspects of the membranes, noting their elasticity and flexibility. It can be seen that the membranes were quite transparent, with an opalescent white color.

The results of the physical tests (Table 2) showed that the control and nanovit membranes had very similar physical characteristics, with no significant differences in terms of mass, thickness, and diameter. The nanovit membrane had a surface that was more oily, compared to the control membrane, which was due to the lipophilic nature of vitamin D_3_.

**Table 2:** Physical characteristics of the control and nanovit membranes.

Membrane	Nanovit	Control
Mass (g)	12.095 ± 1.27	11.30 ± 1.25
Thickness (mm)	3.75 ± 0.35	3.55 ± 0.26
Diameter (mm)	93 ± 1.41	91.5 ± 1.29
Strength (N)	23.7 ± 2.68	13.9 ± 0.98
ℓ0 (mm)	60	60
Δ ℓ (mm)	40.8 ± 2.76	27.1 ± 1.87
ℓ f (mm)	98.8 ± 2.77	85.7 ± 1.87
Area (mm^2^)	80	80
Strength (N/mm^2^)	0.29 ± 0.04	0.17 ± 0.17
Specific deformation (%)	64.7 ± 4.66	42.9 ± 3.18
Young’s modulus (MPa)	0.46 ± 0.02	0.40 ± 0.05
Crushing test (kN/m)	3.16 ± 0.10	3.02 ± 0.10
Tear resistance test (kg f)	15.2 ± 0.66	11.5 ± 1.50

The data obtained in the traction tests indicated that the membrane chemical composition influenced the stretching behavior, with the presence of the vitamin acting to increase the elasticity. The nanovit membrane presented a stretching value of 40.8 mm, with a standard deviation of 2.76 mm, while the value for the control membrane was 27.1 mm, with a standard deviation of 1.87 mm.

The Young’s modulus parameter provides an indication of the intrinsic rigidity of a material^54,55^. The nanovit membrane presented an average value of 0.46 MPa, with a standard deviation of 0.02 MPa, while the average value for the control membrane was 0.40 MPa, with a standard deviation of 0.05 MPa. The higher the Young’s modulus, the greater the resistance of the material to deformation^56^. Similar results were reported by Cinman (2014) and Drago (2014) for latex membranes impregnated with drugs, which presented Young’s modulus values of 0.86 and 0.63 MPa, respectively^57,58^.

Higher deformation percentage was observed for the membrane containing vitamin D_3_ (average of 64.7%). When a stress was applied to the material, it became plastically deformed and did not return to its original shape.

### FT-IR spectroscopic analysis

Analysis using FT-IR can enable the detection of possible changes in the physicochemical properties of the material and interactions among the components^59^. Hence, spectra were obtained for the nanocellulose, vitamin D_3_, and the control and nanovit membranes.

The FT-IR spectrum for the nanocellulose showed two main absorption regions, with one in the low wavenumber region (800–1800 cm^−1^) and the other at higher wavenumbers (2700–3800 cm^−1^). A band at 1633 cm^−1^ was characteristic of lignin and cellulose, attributed to the intramolecular hydrogen bonds of lignin, and vibrations of C–H and C–O in the cellulose polysaccharide rings. A band at 3286 cm^−1^ corresponded to –OH stretching^60,61^.

The vitamin D_3_ spectrum presented a band at 3317 cm^−1^, attributed to –O–H hydrogen bonds, while bands at 2972 and 2891 cm^−1^ corresponded to –C–H stretching. A band at 1377 cm^−1^ corresponded to angular deformation of geminal dimethyl, while a band at 880 cm^−1^ could be attributed to vibration of C=CH_2_. The observed bands were in agreement with the literature^62,63^.

**Figure 3:**
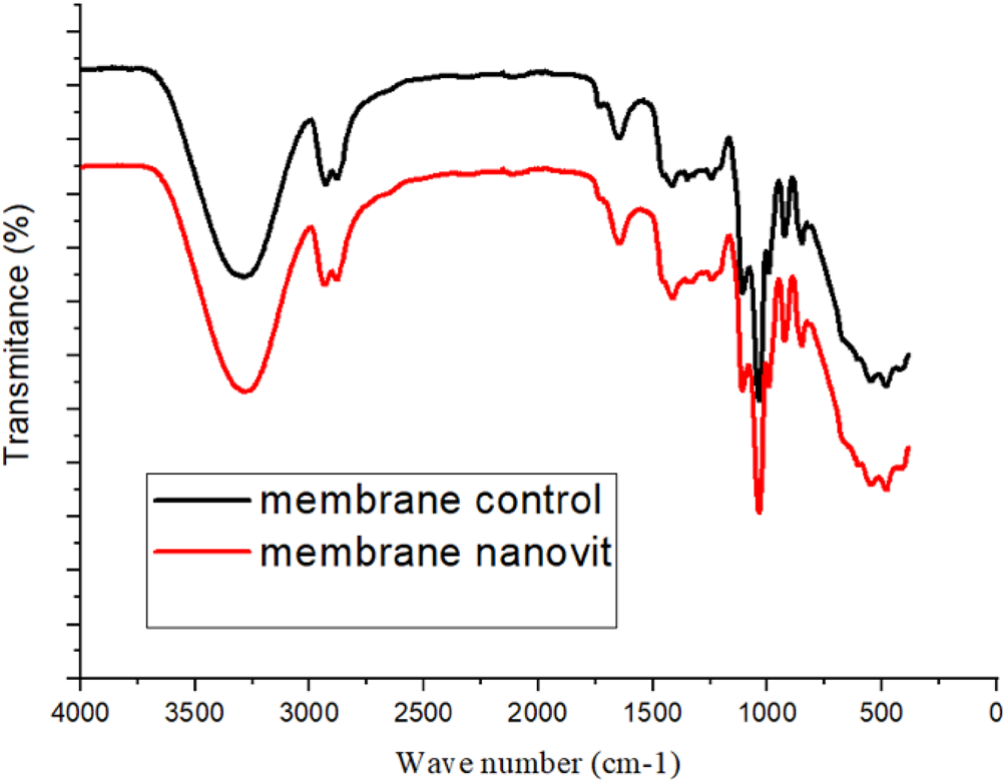
FT-IR spectra for the control and nanovit membranes.

It could be seen from comparison of the FT-IR spectra for the control and nanovit membranes (Fig. 3) that there were no structural alterations of the membrane components. There was no appearance of new bands or other spectral alterations, indicating the absence of cross-linking or formation of new bonds in the membrane^64^. If such bonds were formed, they would be expected to influence the process of sustained release of vitamin D_3_^58^.

The FT-IR results showed that all the components presented –OH stretching signals. The bands at 3286 and 1633 cm^−1^ in the NC spectrum were also present in the spectra for the control and nanovit membranes. The bands at 1377, 1043, and 881 cm^−1^ in the vitamin D_3_ spectrum were also present in the nanovit membrane spectrum.

It should be stressed that no new bands were detected in the FT-IR spectra of the membranes, confirming the integrity of the drug in the membrane. Its functional groups were maintained, with no induction of cross-linking, indicating that this system possessed the necessary characteristics to function as a carrier for this active agent^65^.

### Characterization of the nanocellulose and the membranes by high-resolution electron microscopy (FEG-SEM)

The FEG-SEM images enabled visualization of the topography of the membranes, providing a detailed evaluation of the surface of the material and assessment of the dimensions of the NC used in the synthesis of the membranes.

**Figure 4:**
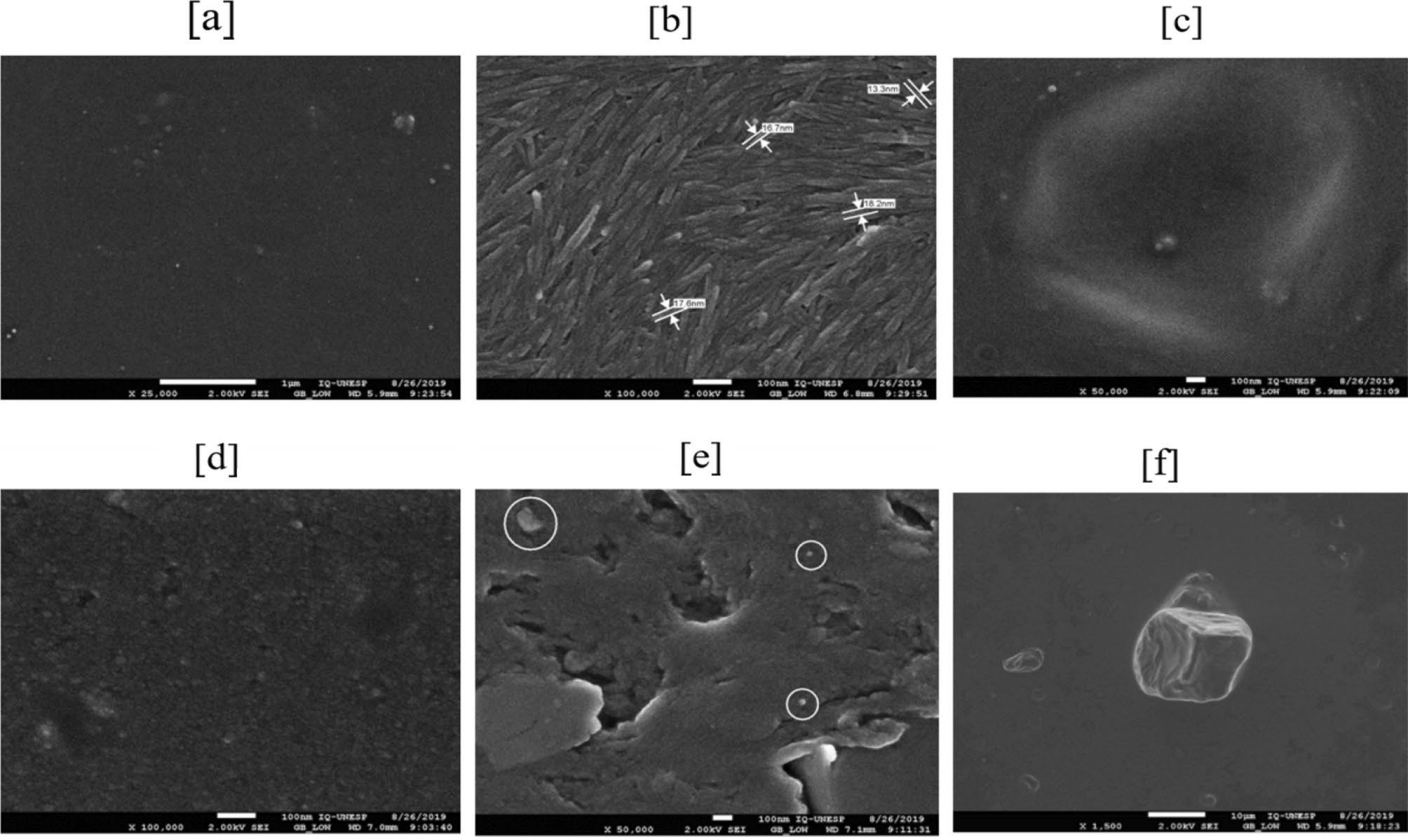
Scanning electron microscopy (FEG-SEM) images of (**a**) the control membrane-100,000 ×, (**b**) the suspension of crystalline nanocellulose—100,000 ×, (**c**) micelles in nanovit membrane—50,000 ×, (**d**) the membrane with addition of vitamin D3—25000x, (**e**) the membrane after release in solution—50,000 ×, and (**f**) micelles and crystals of vitamin D3—1500 ×.

The control membrane presented continuity, homogeneity, absence of porosity, and light surface texture (Fig. 4a). There were no signs of aggregation of polymers visible on the membrane surface. This is a very important feature for this type of composite, since it maximizes the effect of incorporation of nanoparticles in the polymer matrix^66^. Similar results have been reported previously for electron microscopy analyses of nanocellulose membranes with characteristics analogous to those obtained here, in terms of topography^26,67,68^.

Figure 4b shows a FEG-SEM image of the NC suspension obtained by acid hydrolysis of the cotton linter. It can be seen that at least one of the dimensions was at the nanometric scale, below 100 nm^69–71^. There was an entangled network of nanocrystals with a high degree of parallel orientation. The NC fibers presented tubular shapes, with widths in the region of 15 nm. It has been reported previously that cellulose nanocrystals obtained from the acid hydrolysis of fibers have the shape of small cylinders or rods^45,72,73^.

Figure 4c shows the presence of micellar structures in the nanovit membrane, as expected since Tween 80 surfactant was added in the membrane synthesis, in order to reduce the surface tension between the components of the formulation^27^. This surfactant has the characteristic of forming micelles in mixtures of oil in water^74^. The image in Fig. 4d, obtained at a magnification of 1500 ×, shows the presence of many micelle-like structures. Figure 4e shows an image of a nanovit membrane after use in the assays of vitamin release in alcoholic solution. After release of the active agent, the membrane presented a porous, heterogeneous, and irregular surface. The presence of vitamin D_3_ crystals can be seen within the bulk membrane. The structure of the membrane was altered, compared to the control membrane, suggesting that release of the drug occurred by a process of erosion, rather than by diffusion.

The image shown in Fig. 4f, obtained at a magnification of 50,000 ×, shows the presence of crystals within the micelles, which could be attributed to vitamin D_3_, since these structures were not observed in the control membrane. There was an absence of agglomerates on the membrane surface, providing further evidence that the NC and PVA matrix could be considered a satisfactory system for the release of vitamin D_3_.

The characteristics of the control and nanovit membranes were consistent with those of polymeric materials with homogeneous topography, presenting absence of porosity, smoothness, continuity, and absence of agglomerates^75–77^. There was a clear difference in surface texture between the control and nanovit membranes, concerning surface oiliness and the presence of micelles. The FEG-SEM images showed the presence of vitamin D_3_ crystals on the surface of the nanovit membrane, indicating that the system could provide rapid release of the drug.

### Drug release assays

The release assays were performed using the control membrane (without addition of vitamin D_3_), the nanovit membrane (with 8000 IU mL^−1^ of vitamin D_3_), and the nanovit super membrane (with 40,000 IU mL^−1^ of vitamin D_3_).

The receptor liquid that showed the best optical response was absolute ethyl alcohol, since use of the solution with 50% ethyl alcohol and 50% alkaline phosphate buffer resulted in interference in the spectrophotometric reading, so it was not possible to perform UV–Vis analysis of these solutions^78–80^. After immersion of the membranes in 200 mL of absolute ethyl alcohol, under constant agitation, 5 mL aliquots were withdrawn at intervals of 60 min for measurement of absorbance^81^.

**Figure 5:**
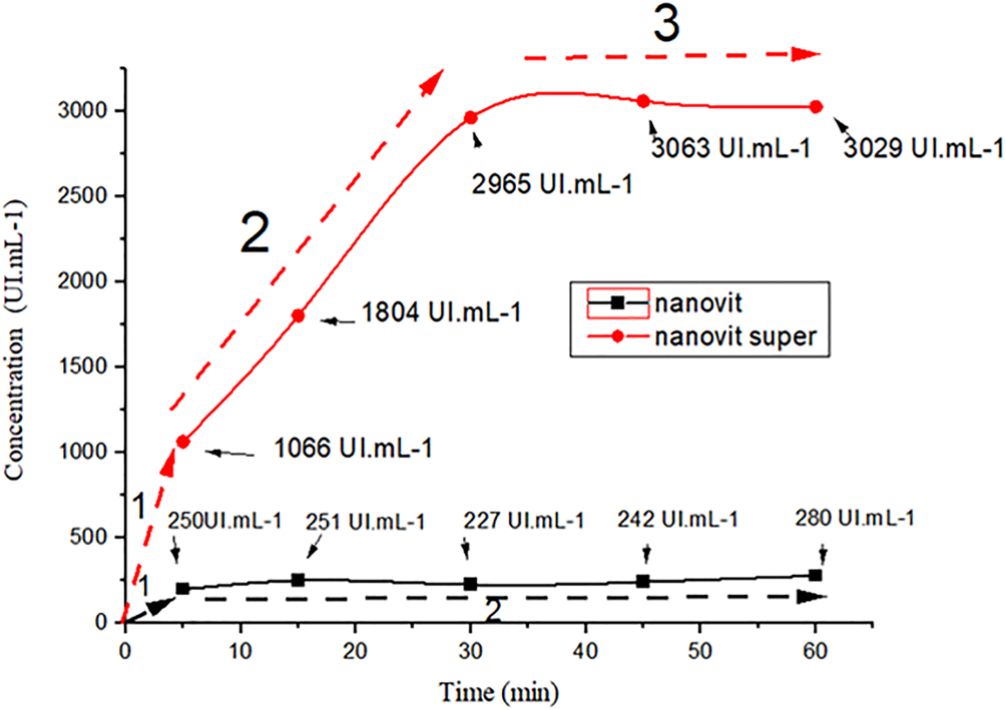
Release of vitamin D_3_ from the nanovit and nanovit super membranes, as a function of time, obtained by absorbance measurements at 265 nm.

Figure 5 shows the concentrations as a function of time, using the control membrane as a reference and obtaining the vitamin D_3_ concentrations in the solutions exposed to the nanovit and nanovit super membranes. For the nanovit membrane, the drug reached the peak concentration rapidly, in 5 min, with a concentration of 250 IU mL^−1^ in phase 1 of the graph. This behavior could be explained by the release of the drug adsorbed on the surface of the membrane, with the concentration values reaching a plateau in phase 2, due to the low concentration of vitamin D_3_^22,82^.

The nanovit super membrane presented 3 distinct phases of vitamin D_3_ release. In phase 1, the release from the surface occurred in 5 min, reaching a concentration of 1066 IU mL^−1^. In phase 2, release between 5 and 30 min was related to the fraction of the drug present within the bulk membrane, obtaining values between 1066 and 2965 IU mL^−1^. In phase 3, the release from the membrane reached a plateau, with a maximum value of 3063 IU mL^−1^^21,83^.

It should be noted that the release from the nanovit membrane occurred rapidly, in 5 min, while the release from the nanovit super membrane lasted 30 min, until saturation of the receptor liquid.

Some groups of formulations were able to release vitamin d3 in a controlled manner, such as microspheres and nanoparticles of hydrophobic alginate derivatives as oral carriers for the sustained release of vitamin D_3_^84,85^. Thus, the development of a crosslinking process, stability tests and in vivo release for this system will be necessary. This work may further extend the in vivo study and provide a reference for the development of a new system to transport vitamin D_3_ in the future.

## Conclusions

Vitamin D_3_ was successfully incorporated in a new system for drug delivery, formed by the association between crystalline nanocellulose, extracted from cotton linter, and polyvinyl alcohol. The “casting” technique used for membrane production was shown to be reproducible, facilitating the production of new membranes.

Analysis using FT-IR spectroscopy demonstrated that there was compatibility among the constituents of the membrane, without the formation of new chemical bonds. The FEG-SEM images showed that the material presented a homogeneous composition, highlighting the presence of drug crystals on the surface and within the membrane. The results confirmed that vitamin D_3_ was incorporated in the membrane, with the interaction enabling fast and progressive release of the drug into the receptor liquid.

Further development of this system should consider the incorporation of other drugs, the use of the membrane in in vivo drug release studies, and the development of a quality control protocol to evaluate the stability of the membrane components.

## Data Availability

All data generated or analysed during this study are included in this published article.

## Abbreviations

NC: Nanocellulose
PVA: Poly(vinyl alcohol)
FT-IR: Fourier transform infrared spectroscopy
UV–Vis: Molecular spectroscopy in the ultraviolet and visible regions
FEG-SEM: Field emission gun scanning electron microscopy
